# Long-term engagement in smoking cessation campaign: A mixed methods randomized trial

**DOI:** 10.1371/journal.pone.0318160

**Published:** 2025-01-30

**Authors:** Robert Schwartz, Tracey Borland, Maaz Shahid, Karen Kuzmich, Michael Chaiton

**Affiliations:** 1 Dalla Lana School of Public Health, University of Toronto, Toronto, Ontario, Canada; 2 Institute of Mental Health Policy Research, Centre for Addiction and Mental Health, Toronto, Ontario, Canada; 3 Canadian Cancer Society, Toronto, Ontario, Canada; NICE: National Institute for Health and Care Excellence, UNITED KINGDOM OF GREAT BRITAIN AND NORTHERN IRELAND

## Abstract

**Introduction:**

A long-term engagement (LTE) intervention was embedded in a social marketing campaign aimed at motivating quit attempts among Canadian adult commercial tobacco users 35 to 64 years of age. The purpose of this study was to examine the effectiveness and appeal of LTE within a marketing campaign.

**Methods:**

3,199 Canadians who smoked cigarettes aged 35–64 recruited using Facebook and Instagram advertisements were randomized into Intervention and Control groups. Over the course of two years, Intervention Group participants received monthly emails connecting them to campaign news and activities, Mini Surveys to inform campaign refinement, feedback opportunities via focus groups and interviews, financial incentives, and proactive knowledge exchange presenting study findings. Both groups responded to a baseline and follow-up surveys every six months.

**Results:**

LTE Intervention Group participants engaged frequently with emails (unique opens, open rates, click rates, and total clicks). Compared with Control Group members, they had significantly higher rates of: unaided and aided campaign awareness; engagement with the social marketing campaign (website visits, social media visits, likes/shares); actions towards making quit attempts; quit attempts. Many participants expressed feelings of motivation, support, and a sense of belonging.

**Conclusions:**

When embedded in a social marketing campaign, meaningful long-term engagement of adults who smoke cigarettes shows promise as an intervention to promote quitting behaviors.

**Implications:**

Active long-term engagement can significantly amplify the effects of social marketing campaigns aimed at promoting quit smoking behaviours among adults. The active engagement approach applied indicates that frequent long-term engagement is more effective when done in a more holistic, empathetic manner. The intervention also allows for real-time learning to support campaign development.

**Trial registration:**

ISRCTN94797633.

## Introduction

The challenges of smoking cessation are well documented. A smoker’s cessation journey typically includes repeated cycles of quit and relapse over the course of many years [1]. Consistent with smoking being a chronic relapsing disease, most smokers want to quit smoking and most do attempt to quit at least once every year [1]. On average, people who smoke make 20 or more quit attempts before succeeding [2]. To address this chronic relapsing disease, long-term engagement (LTE) interventions have shown some promise [3–5]. It is also well documented that social marketing campaigns can effectively yield increases in quit attempts [6–8].

Because many smokers take years to quit, even after they have expressed a strong desire to do so, long-term engagement based on the chronic care model is a promising approach to shortening their cessation journeys [3]. Most smoking cessation interventions are one-off and short-term and fail to address high rates of lapse and relapse [3]. The conceptual framework of LTE posits that engagement over long periods of time, i.e. 12 months or more, will kindle or rekindle latent intentions to quit leading to recycling (additional quit attempts) [3–5]. Additional quit attempts increase the chances of quitting [9] even in the absence of cessation supports (pharmacotherapy and psychosocial counseling). As cessation support increases the likelihood that a quit attempt will succeed, [10] the intervention will promote uptake of pharmacotherapy and psychosocial counseling.

Engagement in tobacco treatment predicts better cessation outcomes. Addiction treatment studies have consistently shown that patients who remain engaged in counselling typically have favourable outcomes during treatment as well as longer-lasting post-treatment benefits [3, 10, 11]. Similarly, a review of studies examining the association of engagement in clinical settings [12] finds that engagement is related to effectiveness. American studies of LTE yielded promising results [3]. In one study, smokers were engaged for 24 months during which they received up to 6 telephone calls in each 6 month interval. More than 60% of participants remained engaged for the full two year period and expressed high levels of satisfaction with their continued engagement [3]. Smokers who received engagement calls had significantly higher rates of cessation than those in the control group [4]. Another study found that smokers who received an average of 16.5 calls over the course of 48 weeks had significantly higher cessation rates than those who received only 2 calls [3–5].

The results of a Canadian LTE trial support the American findings of the positive effects of the engagement process [13]. 1,621 smokers and recent quitters were randomly assigned to an intervention and control group. Intervention group participants received 11 monthly emails with a mini-poll and links to cessation services, while the control group did not receive any emails. Almost all intervention group participants remained subscribed to the monthly emails throughout the 11-month intervention period, most opened the monthly emails and responded to the mini-poll asking about their current smoking. Emails helped participants: think about quitting (41%); stay smoke-free (24%); cut down (23%), increase motivation (23%); make a quit attempt (22%). Nevertheless, fewer than 10% of participants clicked on the links to cessation services and no differences were found between intervention group and control group participants in smoking status and relapse outcomes [13].

Research to date suggests that LTE is a promising intervention, yet it has been tested in only a small number of contexts. Canada’s recent Smoke-Free Curious Campaign (SFC) afforded opportunity to test the effectiveness of providing long-term engagement as part of a social marketing campaign.

SFC was a social marketing campaign created by the Canadian Cancer Society, in collaboration with project partners. Similar campaigns were run in English and in French with appropriate adaptation for the French language campaign. The overall goal of SFC was to motivate quit attempts among Canadian adult commercial tobacco users 35 to 64 years of age. The campaign aimed to increase awareness of the consequences of smoking and the benefits of quitting (or reducing), intentions to reduce/quit smoking and quit attempts. Through a variety of social media and out of home platforms, SFC reached an estimated 5,432,814 Canadians. 14,854 who smoke were provided NRT trial packs and 7,965 participated in Quit and Win contests. This study tests the effectiveness of the long-term engagement used within the campaign.

## Methods (Population, intervention, comparators, outcomes and analysis)

A mixed methods randomized control trial was conducted to assess the effects of incorporating LTE into a social marketing campaign on participant quit smoking journeys. The study protocol is available as a supporting information file (S1 File).

### Population

**Fig 1:**
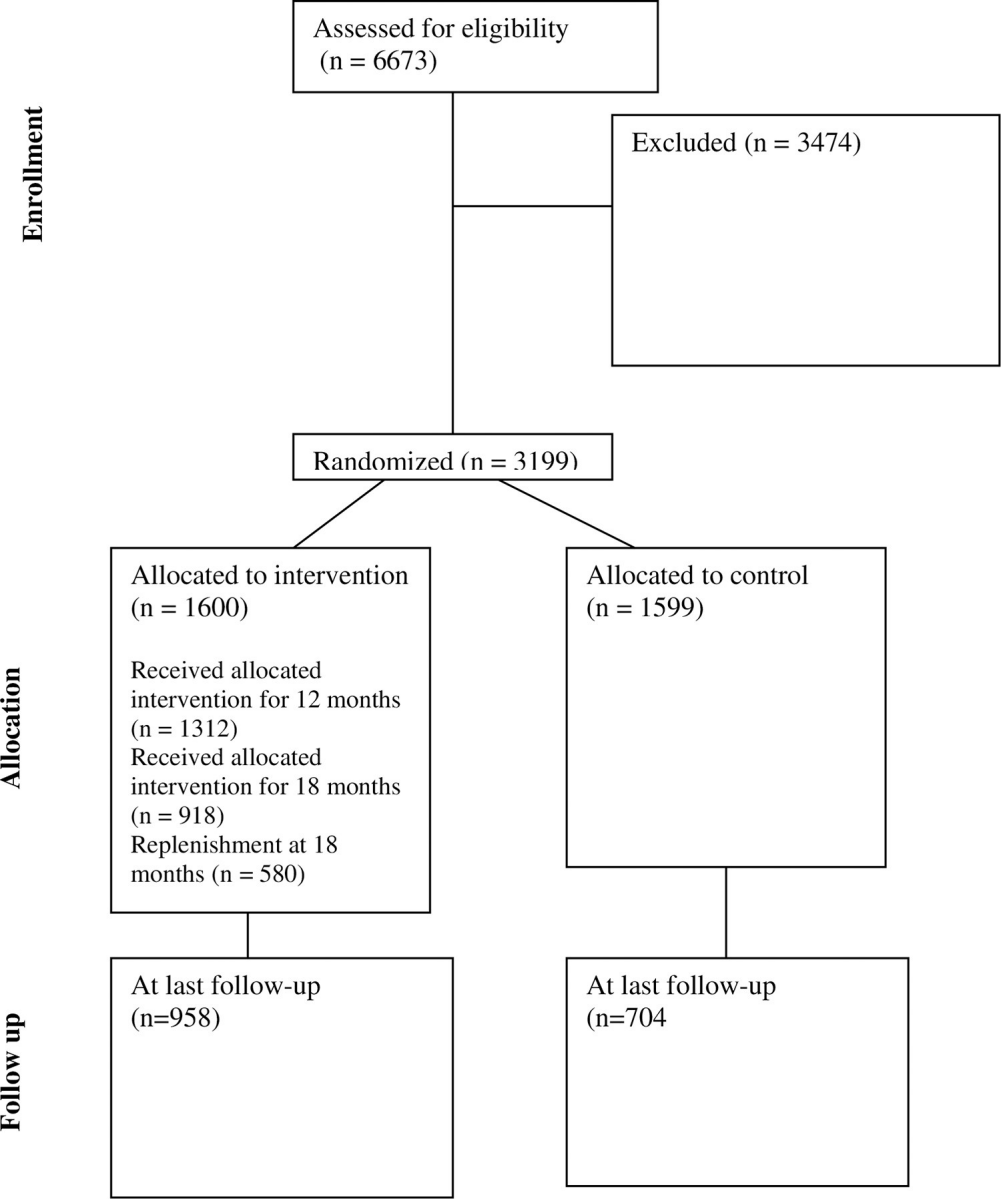
CONSORT diagram.

A cohort of 3,199 participants (35 to 64 years of age) was recruited at baseline using Facebook and Instagram advertisements in late Summer/Fall 2021 (August 2021 to December 2021) (Fig 1). To be eligible, participants needed to be from 35 to 64 years of age, currently smoke cigarettes and live in the province of Ontario or Quebec where the campaign was run. Applicants identified as bots were excluded. Participants received general information about the study and a consent form about the study, follow-up, and their rights to privacy and confidentiality, the right to refuse to answer any question. Consenting participants were able to start the online survey. Participants who did not consent to participate were thanked for their time. Data collection, including follow-up surveys, occurred from September 2021 through December 2023.

Half of the cohort was randomly assigned to the Intervention Group and half to the Comparison Group. The Senior Research Associate, used the Microsoft Excel randomization function, participants were randomly assigned to 10 groups. Those assigned to even number groups (2, 4, 6, 8 and 10 were assigned to the intervention group and those assigned to odd number groups (1, 3, 5, 7 and 9) were assigned to the control group. Participants were not informed of their assignments to intervention or control groups. The Research Officer responsible for data collection was aware of group assignment as they sent monthly emails to intervention group members only, as specified below.

### Intervention

Intervention group members were actively engaged with the campaign for two years through various activities. They received emails in 20 of the 24 months connecting them to campaign news and activities, five Mini Surveys to inform campaign refinement, opportunities to give additional feedback via focus groups and interviews, and proactive knowledge exchange materials presenting study findings (i.e., infographics). Emails were sent using Mailchimp and espoused the tone of empathy and compassion, which were central traits of the campaign. Intervention Group participants received an additional $160 and $70, in the first and second years respectively, for their participation. Incentives were distributed in smaller amounts, and often coincided with a Mini-Survey. At 18th months follow-up, 679 new participants were on-boarded into the Intervention Group for replenishment. Participants received a $10 CAD e-gift card administered via Giftbit for each survey completed. E-gift cards were sent prior to the surveys as a form of pre-payment. Surveys were administered via the data capture tool Redcap and participants received up to 4 weekly reminders per survey.

The five Mini Surveys collected feedback on a variety of topics. Feedback often focused on the knowledge needs of the Canadian Cancer Society. In Year one, the surveys asked for feedback on: 1) the campaign’s initial direction, name and creative 2) social media preferences 3) the experience as an Intervention Group member. In year 2, the surveys explored participants’ 4) perceptions of quitting milestones and preferences for sharing milestones and contests and 5) feedback of the new #IamQuitting Milestone Contest. A bonus survey also occurred in Year 2 to obtain feedback about potential next steps for Smoke Free Curious.

Table 1 illustrates intervention group participation over the course of the study period. The first email to the English-speaking Intervention Group was successfully delivered in March 2022 to 1197 participants. Retention over the first year was high such that the March 2023 email was successfully delivered to 1,137 participants. Phase 2 of the LTE intervention started in May 2023 with successful deliveries to 743 participants who volunteered to continue in the study for an extra year. This number remained steady through October 2023 when 456 new participants were added from new recruitment for replenishment. In November, after replenishment, the English group included approximately 1,189 participants. Total unsubscribes were minimal.

**Table 1:** Intervention group participation over time.

	English	French
**Spring 2022**	**1,197**	**387**
**Spring 2023**	**1,137**	**175**
**Summer 2023**	**743**	**175**
**November 2023 w/replenishment**	**1,189**	**299**

Similarly, the first email, in May 2022, to the French-speaking Intervention Group was sent to 387 French-speaking participants. From May 2023 to Oct 2023, the email was sent to approximately 175 intervention group participants (those who agreed to stay in the study for the extra year). In November, after replenishment, the French group included approximately 299 participants. Total unsubscribes were minimal.

### Comparators

Control group members received no proactive engagement. They may have been exposed to Campaign messaging as would any other Canadian adult person who smokes. Both Intervention Group and Comparison Group participants received invitations to complete a baseline survey (prior to campaign launch) and follow-up surveys at 6-month intervals (conducted at 6-, 12-, 18-, and 22-months post-campaign launch). The French campaign was launched three months after the English campaign. Thus, French follow-up surveys took place at 3-, 9-, 15-, and 19-months post-campaign launch.

### Outcomes

The baseline survey gathered data about participants’ demographic information, smoking and quitting behaviour, experience with quit smoking resources and ideas and suggestions for a future cessation campaign to help adults make quit attempts. Follow-up surveys sought to capture data on participants’ awareness, experiences with the campaign and behavioral changes related to smoking. Specifically, surveys included questions related to knowledge (i.e., harms, benefits of quitting and cessation supports available), campaign awareness (aided and unaided), commercial tobacco use behaviour (e.g., smoking status, heaviness of smoking, quit attempts, intentions to quit, confidence in quitting etc…), campaign satisfaction/resonance and perceived campaign impacts. Socio-demographic data were also collected (i.e., age, gender, race, geography).

In addition to the LTE intervention trial, the study included standard approaches to evaluating social marketing campaigns: web analytics, surveys, focus groups and interviews (results not reported here). Research was conducted in English and French and took place between August 2021 and March 2024 with University of Toronto REB approval (41076). This study was not registered as a clinical trial prior to enrolment of participants as we did not realize that this study would be considered to be a clinical trial. It has subsequently been registered (ISCTRN46203). The authors confirm that all ongoing and related trials for this drug/intervention are registered.

### Analysis

Intervention group member responses to follow-up surveys were compared with those of control group members to ascertain similarities and differences in campaign awareness, engagement with the campaign components, accessing cessation resources, campaign perceptions, quitting and smoking behaviours. Intervention group engagement with monthly emails was measured with simple email analytics (unique opens, open rates, click rates, and total clicks). The statistical analyses were conducted using STATA version 15.1. Statistical significance was assessed using Pearson’s chi-square test (two-tailed) and an acceptable significance level of 0.05.

### Missing data

Between the baseline population and the second follow up at 12 (English cohort) and 9 (French cohort) months, there was an attrition of just under 50% of the population–meaning they did not respond to either the first or second follow-ups. Intention to Treat analysis was not conducted as intervention group non-respondents would also not have benefitted from the monthly emails. There was a fairly high response rate when it came to items in the questionnaire–with the lowest amount being around 75% of respondents answering a specific question. With this context in mind, a complete case analysis strategy was used in that respondents had to have completed a follow-up questionnaire to be included in the analysis.

## Results

A total of 2,420 participants completed the baseline English survey (Table 2). The majority of participants are located in Ontario (53.1%), identify as a woman (59.1%), identify as White–North American (67.1%), and speak English at home (93.1%). The majority of participants indicated smoking every day (94.7%), with 50.9% of participants smoking 11–20 cigarettes per day. Further, 50.5% indicated smoking more due to COVID-19. Of 2,412 participants, 21.8% plan to quit smoking in the next two months, 43.3% plan to quit smoking in the next six months, and 28.3% plan to quit smoking in the future beyond six months.

**Table 2:** Demographic characteristics of English and French evaluation participants, baseline, 18 (English)/15 (French) and 22 (English)/19 (French)- months follow-up.

	English			French		
	Baseline	18 months	22 months	Baseline	15 months	19 months
	N = 2420	N = 1752	N = 1306	N = 780	N = 565	N = 355
**Age**						
<40	27.1%	25.2%	26.0%	16.3%	22.7%	20.3%
40–49	34.2%	35.7%	35.4%	32.2%	37.0%	38.3%
50–59	24.8%	26.4%	26.6%	33.7%	29.4%	32.7%
60+	13.9%	12.7%	11.9%	17.8%	11.0%	8.7%
**Gender**						
Woman	59.1%	57.6%	59.5%	54.7%	60.2%	55.8%
Man	39.0%	40.5%	38.2%	40.3%	36.3%	40.1%
Gender non-binary	0.4%	0.3%	0.5%	--	0.2%	--
Two-Spirit	0.4%	0.3%	0.5%	0.3%	--	--
Gender fluid	0.4%	0.5%	0.4%	1.3%	0.9%	1.0%
Genderqueer	0.3%	0.4%	0.4%	0.6%	0.5%	2.0%
I do not know	0.3%	0.1%	0.1%	1.5%	0.9%	1.0%
Prefer not to answer	0.3%	0.5%	0.5%	1.7%	0.9%	1.0%
Prefer to Self-Identify	0.1%	0.1%	0.2%	0.3%	0.2%	--
**Province/Territory**						
Ontario	53.1%	56.7%	56.1%	0.6%	0.7%	0.8%
Alberta	11.0%	10.2%	10.5%	--	--	--
British Columbia	10.1%	8.9%	10.0%	0.1%	--	--
Nova Scotia	6.9%	5.5%	5.4%	--	--	--
Quebec	4.5%	5.8%	5.1%	97.7%	98.4%	98.0%
New Brunswick	4.0%	3.5%	3.6%	1.2%	0.9%	1.1%
Manitoba	3.8%	3.6%	3.6%	0.1%	--	--
Saskatchewan	3.0%	2.5%	2.5%	0.1%	--	--
Newfoundland and Labrador	2.6%	2.3%	2.4%	--	--	--
Prince Edward Island	0.7%	0.6%	0.6%	--	--	--
Yukon	0.2%	0.2%	0.2%	--	--	--
Northwest Territories	0.2%	0.2%	0.1%	0.1%	--	--
**Ethno-racial background**						
White—North American	67.1%	67.2%	69.7%	75.9%	79.8%	77.2%
White—Other	20.0%	20.0%	16.9%	15.9%	15.0%	14.2%
First Nations	3.8%	3.4%	3.7%	1.2%	1.7%	2.5%
Mixed heritage	3.6%	3.7%	3.2%	0.5%	0.5%	0.5%
Indigenous	2.7%	2.4%	2.6%	1.3%	1.4%	1.5%
Métis	2.6%	2.8%	2.6%	1.5%	1.2%	1.5%
Prefer not to answer	1.6%	1.2%	1.1%	2.6%	0.5%	1.5%
Black–Caribbean	1.0%	0.9%	0.9%	0.1%	0.5%	0.5%
Middle Eastern	0.9%	1.1%	1.1%	--	--	--
Latin American	0.9%	0.7%	1.1%	0.4%	0.7%	--
I do not know	0.8%	0.7%	0.4%	1.4%	1.8%	--
Southeast Asian	0.7%	0.9%	1.0%	0.1%	--	--
East Asian	0.7%	0.7%	0.8%	--	--	--
South Asian	0.6%	0.9%	0.7%	--	--	--
Black–North American	0.5%	0.6%	0.5%	--	--	--
Inuk/Inuit	0.4%	0.3%	0.3%	--	--	--
Black–African	0.3%	0.5%	0.3%	0.1%	--	--
Prefer to self-identify	0.2%	0.2%	0.3%	0.4%	0.2%	--

A total of 779 participants completed the baseline French survey (Table 2). The majority of participants are located in Quebec (97.7%), identify as a woman (54.8%), identify as White–North American (75.9%), and speak French at home (96.9%).The majority of participants indicated smoking every day (95.6%), with 49.9% of participants smoking 11–20 cigarettes per day. Further, 35.4% indicated smoking more due to COVID-19. Of the 779 participants, 19.6% plan to quit smoking in the next two months, 31.5% plan to quit smoking in the next six months, and 19.2% plan to quit smoking in the future beyond six months.

At baseline, intervention and control group participants in both the English and French language samples were similar across demographic and smoking/quitting measures (Table 3).

**Table 3:** Intervention and control group participant characteristics at baseline.

	English		French	
	Intervention	Control	Intervention	Control
	N = 1148	N = 1204	N = 373	N = 384
**Age**				
**<40**	**34.6%**	**34.5%**	**15.2%**	**16,0%**
**40–49**	**24.1%**	**24.7%**	**30.7%**	**34.9%**
**50–59**	**13.2%**	**14.0%**	**37.2%**	**30.2%**
**60+**	**28.0%**	**26.8%**	**16.0%**	**19.6**
**Gender**				
**Woman**	**58.5%**	**59.8%**	**52.9%**	**57.1%**
**Man**	**39.7%**	**39.1%**	**43.0%**	**38.1%**
**Province/Territory**				
**Ontario**	**52.8%**	**53.6%**	**0.3%**	**1.0%**
**Quebec**	**4.2%**	**4.6%**	**98.7%**	**96.9.%**

### Engagement with monthly emails

**Fig 2:**
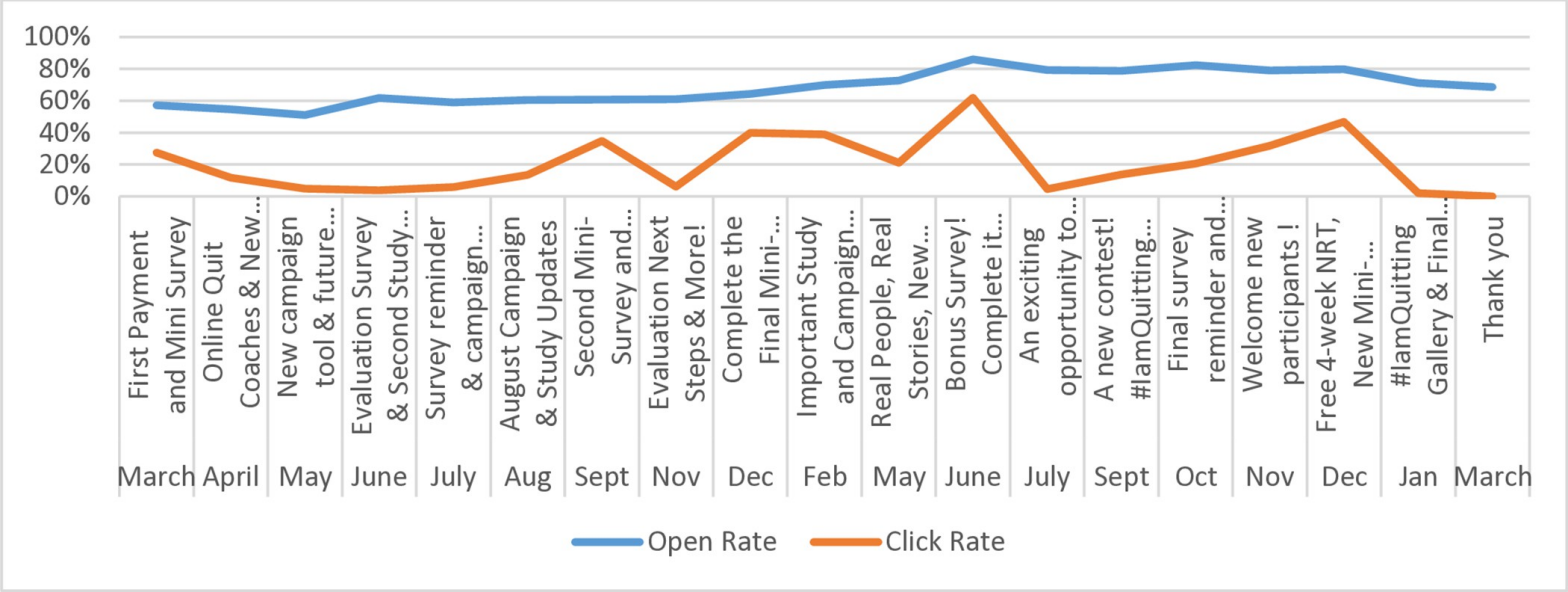
Email open and click rates (%), English Intervention Group, March 2022-March 2024.

Email analytics (Figs 2 through 5) indicate consistently high engagement across both English and French email campaigns, although fluctuations in metrics were observed (i.e., unique opens, open rates, click rates, and total clicks). Unsubscribes were low throughout the email campaigns, suggesting satisfaction with being part of the Intervention Group. Further, some emails appear to have had higher engagement than others, indicating varying levels of audience interest and interaction with specific content. Emails containing incentives, such as surveys, contests, NRT or payment reminders, appear to have higher click rates and total clicks. Every email had the opportunity for participants to click on a link.

**Fig 3:**
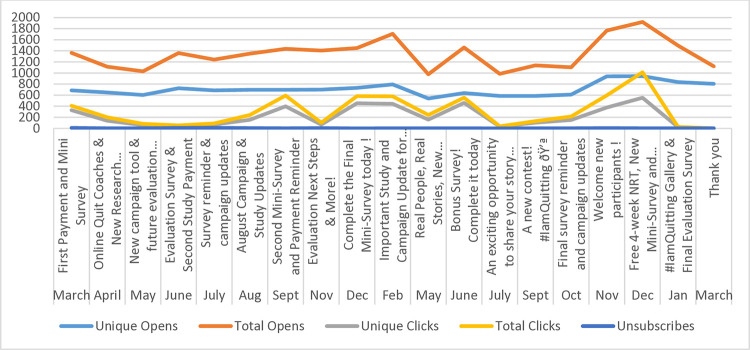
Opens, clicks and unsubscribes, English Intervention Group, March 2022-March 2024.

**Fig 4:**
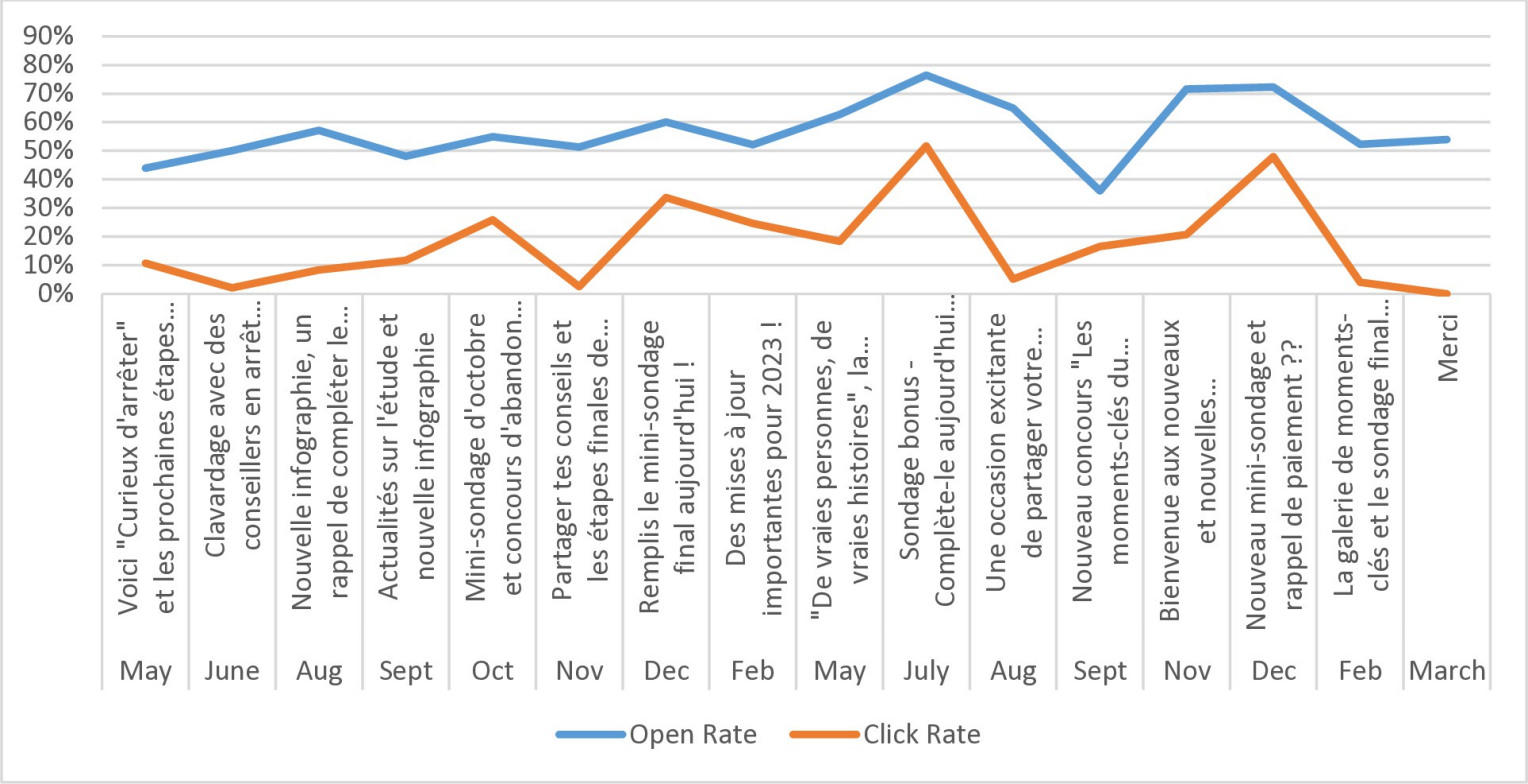
Email open and click rates (%) French Intervention Group, March 2022-March 2024.

**Fig 5:**
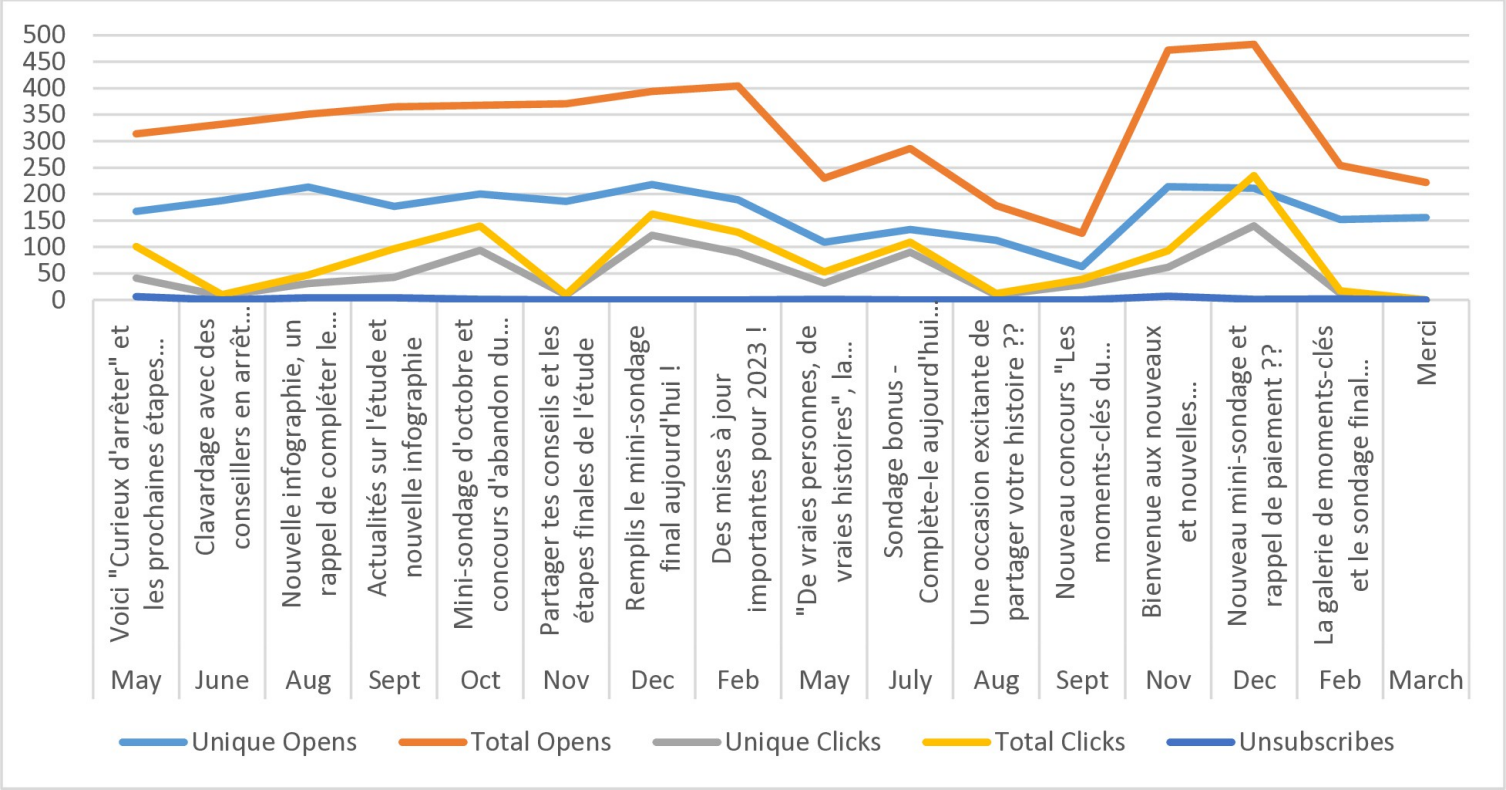
Email open and click rates, French Intervention Group, March 2022-March 2024.

### Awareness of the campaign

Both English and French language Intervention Group participants had substantially higher rates of unaided awareness of the campaign than Control Group participants across timepoints (Table 4). At the last timepoints (22 months for English and 19 months for French) 70.3% (English) and 65.9% (French) of Intervention Group participants were aware of the campaign compared with 37.2% (English) and 28.9% (French) of Control Group participants. Similarly, at 12-months follow-up, English Intervention Group participants had significantly higher levels of campaign awareness compared to the Control Group (62.3% vs 33.5%, p< 0.001). Further, a greater proportion of Intervention Group members had unaided unawareness (27.2% vs 12.3%, p< 0.001), meaning they were able to identify the campaign without being provided with a verbal or image hint.

**Table 4:** Awareness of SFC campaign between participants in control and intervention groups.

**All participants**	**English**		**P-value** *
	**Control at baseline**	**Intervention at baseline**	
	**(n = 1204)**	**(n = 1148)**	
Aware of campaign @ 12 months **(n = 642)**	33.5%	62.3%	p < 0.001
Aware of campaign @ 18 months **(n = 550)**	18.4%	39.9%	p < 0.001
Aware of campaign @ 22 months **(n = 743)**	37.2%	70.3%	p < 0.001
	**French**		
	**Control at baseline**	**Intervention at baseline**	
	**(n = 384)**	**(n = 373)**	
Aware of campaign @ 9 months **(n = 102)**	15.5%	48.3%	p < 0.001
Aware of campaign @ 15 months **(n = 137)**	15.2%	35.3%	p < 0.001
Aware of campaign @ 19 months **(n = 170)**	28.9%	65.9%	p < 0.001

*Pearson’s chi-square test was used

### Engagement with campaign components

Intervention and control Group participant engagement with campaign components are compared in Table 5. Among the English 12-month and 22-month samples, a significantly greater proportion of Intervention Group than Control Group members had:

- visited the SFC website (69.1% vs. 43.7%, at 12 month and 79.9% vs. 58.3%, 65.03at 22 month)
- visited SFC social media (56.4% vs. 45%, 35.2, at 12 month and 67.7% vs. 51.0%. at 22 month)
- liked or shared SFC’s social media posts (25.3% vs. 15.6%, at 12 month and 31.4% vs. 24.8%,at 22 month).

**Table 5:** Comparison of intervention and control groups on engagement, accessing resources, quitting and smoking behaviours.

ENGLISH	12 Month			22 Month		
	Intervention (%)	Control (%)	Significant	Intervention (%)	Control (%)	Significant
***Engagement with Campaign Components***						
**Visited SFC Website**	**69.1**	**43.7**	**Yes**	**79.9**	**58.3**	**Yes**
**Visited SFC Social Media**	**56.4**	**45.0**	**Yes**	**69.7**	**51.0**	**Yes**
**Liked/Shared SFC Social Media Post**	**25.3**	**15.6**	**Yes**	**31.4**	**24.8**	**Yes**
***Accessing Resources and Campaign Perceptions***						
**Increase Knowledge about Community Smoking Resources**	**21.2**	**14.7**	**No**	**27.7**	**19.4**	**Yes**
**Increase Knowledge about Online Quit Smoking Resources**	**33.4**	**19.6**	**Yes**	**38.7**	**25.4**	**Yes**
**Harms & Risks of Smoking**	**35.0**	**27.7**	**No**	**35.6**	**24.9**	**Yes**
**Different Ways Available to Quit Smoking**	**30.9**	**22.3**	**Yes**	**36.7**	**26.9**	**Yes**
***Quitting Behaviour***						
**Past 6 Month Quit attempt (1 or more)**	**61.9**	**52.5**	**Yes**	**57.6**	**43.9**	**Yes**
**Quit Attempt Helped by Campaign**	**56.1**	**45.7**	**Yes**	**61.0**	**44.5**	**Yes**
**No Campaign Inspired Quit Actions**	**31.1**	**16.7**	**Yes**	**39.4**	**35.8**	**No**
***Smoking Behaviour***						
**Daily Smoking**	**64.6**	**69.7**	**Yes**	**61.3**	**63.8**	**Yes**
**< 10 Cigarettes per Day**	**31.1**	**24.1**	**Yes**	**30.0**	**28.5**	**No**
**FRENCH**	**9 Month**			**19 Month**		
	**Intervention (%)**	**Control (%)**	**Significant**	**Intervention (%)**	**Control (%)**	**Significant**
***Engagement with Campaign Components***						
**Visited SFC Website**	**73.9**	**36.8**	**Yes**	**70.2**	**41.5**	**Yes**
**Visited SFC Social Media**	**64.3**	**42.1**	**No**	**62.8**	**58.5**	**Yes**
**Liked/Shared SFC Social Media Post**	**40.5**	**21.1**	**No**	**36.4**	**26.4**	**No**
***Accessing Resources and Campaign Perceptions***						
**Increase Knowledge about Community Smoking Resources**	**21.2**	**14.7**	**No**	**27.7**	**19.4**	**Yes**
**Increase Knowledge about Online Quit Smoking Resources**	**33.4**	**19.6**	**Yes**	**38.7**	**25.4**	**Yes**
**Harms & Risks of Smoking**	**35.0**	**27.7**	**No**	**35.6**	**24.9**	**Yes**
**Different Ways Available to Quit Smoking**	**30.9**	**22.3**	**Yes**	**36.7**	**26.9**	**Yes**
***Quitting Behaviour***						
**Past 6 Month Quit attempt (1 or more)**	**46.1**	**30.5**	**Yes**	**54.3**	**33.9**	**Yes**
**Quit Attempt Helped by Campaign**	**47.0**	**21.1**	**Yes**	**41.7**	**68.6**	**Yes**
**No Campaign Inspired Quit Actions**	**93.7**	**84.9**	**No**	**28.0**	**39.4**	**No**
***Smoking Behaviour***						
**Daily Smoking**	**72.4**	**74.2**	**No**	**71.9**	**64.7**	**No**
**< 10 Cigarettes per Day**	**15.5**	**20.7**	**No**	**18.2**	**28.6**	**No**

Significant findings for the French 9-month and 19-month samples were limited to the website, whereby a greater proportion of Intervention Group vs. Control group respondents had visited the website (73.9 vs 36.8%, for 9 months and 70.2% vs 41.5%, for 19 months).

### Accessing resources and campaign perceptions

At 12- and 22-months follow-up, a significantly greater proportion of English Intervention Group than Control Group respondents said that the campaign had increased, ‘to a great extent’ their knowledge about online quit smoking resources (33.4% vs 20%, for 12 months and 38.7% vs 25.4%, for 22 months) and different ways available to quit smoking (30.9% vs 22%, for 12 months and 36.7% vs 26.9%, (Table 5).

This was similar for the French sample at 9 months, where a significantly greater proportion of Intervention Group respondents said that the campaign increased ‘to a great extent’ their knowledge about online quitting resources (33.4% vs 19.5% at 9 months and 38.7% vs 25.4% at 29 months) and about different ways available to quit smoking (30.9% vs 22.3% at 9 months and 6.7% vs 26.9% at 19 months).

### Quitting behaviour

A greater proportion of both English and French Intervention Group members had made one or more quit attempts vs Control Group members at 12- and 9-months follow-up, respectively (EN: 61.9% vs 52.5%,; FR: 46.1% vs. 30.5) (Table 5). This was also seen at the 22- and 19- month follow-ups as well (EN: 57.6% vs 43.4; FR: 54.3% vs. 33.9%,).

Among participants who had made a quit attempt in the past 6 months, greater proportions of both English and French Intervention Group respondents said that the campaign helped them make a quit attempt vs their respective Control Groups (EN: 56.1% vs. 45.7%, FR: 47.0% vs 21.1% at 12 and 9 months respectively)).

When asked if and how the campaign had inspired them, a greater proportion of English Intervention Group members noted being inspired to take a quit support action as listed below. In comparison with the Intervention Group, a greater proportion of Control Group respondents said that the campaign did not inspire them to do anything (31.1% vs 16.7%,)–only significant at the 12-month follow-up (Table 5). At 12 month follow-up English language Intervention Group participants were significantly more likely than Intervention Group participants to have taken the following actions (data not shown):

- Order a Free NRT Trial pack (38.5% vs. 20.9%; p≤0.001).
- Chat with friends or family about quitting smoking (48.1% vs. 38.2%; p = 0.022).
- Look up quit smoking support in your community (25.7% vs 18.3%, p = 0.04).
- Talk to a health professional (31% v 22.5%, p = 0.03).
- Sign up for the First Week Challenge (22.8% vs. 15.59%; p<0.001).

A significantly greater proportion of French Intervention Group respondents had used NRT (14.3% vs 6.8%, p = 0.047 at 9 months and 13.9% vs 7.2%, p = 0.047 at 19 months) or E-cigarettes (12.5% vs 5.1%, p = 0.034 at9 months and 16.3% vs 9.0%, p = 0.045 at 19 months) in the past 6 months.

### Smoking behaviour

At 12 months, a significantly smaller proportion of English Intervention Group respondents reported smoking every day (64.6% vs 69.7%, p = 0.008) and a greater proportion smoked less than 10 cigarettes a day compared to the Control Group (31.1% vs 24.1%, p = 0.007). Similarly at 22 months, a slightly smaller proportion of English Intervention Group respondents reported smoking every day (61.3% vs 63.8%, p<0.001) and not much difference was seen in the proportion who smoked less than 10 cigarettes a day compared to the Control Group (30.0% vs 28.5%, p = 0.610) (Table 5).

In the French sample there were no significant differences in smoking behaviours between Intervention and Control Group participants.

### Role of LTE in quit smoking journeys

The third mini survey (December 2022) and evaluation interviews (February 2023 and 2024) provide insight into the role that the LTE Intervention played in participant quit smoking journeys.

Overall, the LTE Intervention was well-received by participants, with many expressing gratitude for the opportunity to be part of it. Ninety-five percent (95%) of mini-survey participants were satisfied with being an Intervention Group member, with many attributing their success in quitting or reducing smoking to factors such as financial incentives, community support, self-reflection, and accountability. Further, approximately 3 out of 4 participants (76.5%) reported that being in the Intervention Group helped them achieve a quit smoking goal. Mini survey qualitative findings revealed that being part of the Intervention Group led to a reduction in smoking frequency, a sense of community and support, self-reflection, and accountability. The monthly emails also helped to keep the concept of quitting smoking at the front of one’s mind.

…It keeps things conscious; right, like its like forefront now it’s like oh, yes for me, it’s about accountability; right. And it’s about sort of okay, like I’m getting these emails and it’s not a bad thing. It’s a good thing. It’s like a reminder to be like hey, you know it’s time to reset and time to get back onto the website and you know figure out maybe some things that you can do that might help and so I think it’s just, yeah, it’s sort of putting this back into your mind again like okay, it’s time to refocus a little bit and maybe if it’s I am not smoking cigarettes anymore which is what this site has helped me out with-

- Ontario Participant

The interviews further highlighted the positive influence of the LTE intervention in reminding participants of their quit smoking goals, providing resources and support, and encouraging them to stay committed to quitting. Participants again appreciated the financial incentives, particularly gift cards, which motivated them to make quit attempts and complete surveys.

"*Well, no. It [gift cards] are [useful] on days like today I’m in between paydays and I’ve got nothing for supper.”*

- Nova Scotia Participant

“..*it [gift cards] gives me like a kind of you know*, *it is an impetus you know it is an encouragement know*.*"*

- Alberta Participant

The surveys themselves were seen as beneficial for tracking progress, serving as an accountability mechanism and increasing awareness of smoking habits. Some really enjoyed providing their input into campaign development and refinement.

“*it’s like a kind of a tracking my own, my own progress you know like where I am standing."*

“*I got to have my input*. *[Laugh] Oh*, *I know I’m not the only one that has tried and that has done most of these tips like the gum chewing and other things but [Pause] I guess maybe if enough people speak-up they’ll do something*.*”*

Further, many participants expressed feelings of motivation, support, and a sense of belonging through the monthly emails, surveys, and encouragement provided by the study. For some, the LTE Intervention served as a reminder of their purpose to quit smoking and provided them with tools and resources to aid in their cessation efforts. Participants appreciated feeling part of something and being rewarded for their achievements, which boosted their morale and determination. However, despite these positive experiences, some participants still struggled to quit entirely, citing financial constraints or the need for additional support. Nonetheless, the Intervention Group appeared to foster an environment free from judgment, helping participants to take control of their smoking habits and focus on their health.

“Every smoker knows that smoking isn’t good for his health, but with monthly e-mails, surveys and even giving money for participating, I told myself: look, people are caring for you and even pay you to quit. So follow your purpose in your own way and quit!”

“Doing these surveys and staying involved gives me motivation and the feeling that I am not alone in this very difficult journey. I can see the light at the end of the tunnel. It’s a little dim right now, but I know it’s there, just have to stay positive and be strong and do not give up.. It’s quite the uphill battle.”

“The material was encouraging and actually was like a reminder each time I would get an email. I haven’t been successful at quitting but I did make 2 attempts and will try again”

“It became a source of motivation. I had the feeling of being part of something. I loved being rewarded for my achievements.”

Overall, the LTE Intervention facilitated a supportive community that motivated participants to persevere in their quit smoking journey.

## Discussion

Evidence presented here demonstrates that long-term, frequent and active engagement of participants was highly effective. Intervention Group participants were generally very appreciative of the monthly emails, chances to contribute via quarterly short surveys and the compensation provided them for participating in the surveys. These surveys were key in providing timely feedback on the campaign and contributed to its ongoing improvement. Moreover, Intervention Group participants took substantially and significantly more action to change their smoking behaviours with less than 10% not taking any action at all.

These findings add to the relatively sparse literature about the effect of LTE. While previous LTE cessation studies found that monthly telephone calls were highly effective [3–5], this is the first study to demonstrate the effects of LTE through monthly emails and in the context of a social marketing campaign. The high levels of engagement and actions taken to decrease smoking, suggest that engaging, monthly emails over an extended period of time may be a low-cost and highly effective addition to existing cessation treatments which typically end after a small number of contacts over a relatively short period of time. However, the generalizability of results to contexts other than being part of a social marketing campaign should be further explored.

These findings suggest that social marketing campaigns aimed at promoting smoking cessation for adults would benefit from incorporating active long-term engagement of participants. The nature of the intervention would limit participation to those willing to provide contact information (i.e. email address) and to receive frequent correspondence. Further research is needed to understand whether financial incentives are crucial to success and to determine the level of compensation needed. While it may be feasible to provide reasonable incentives to a small proportion of the target population of a social marketing campaign, it would not likely be realistic to make this offer to more than a few thousand participants.

There are a number of limitations to this study. The intervention was targeted to Canadian adults aged 35–64 and other demographic groups may have different perspectives on long term engagement. As with many self-report studies, there is a risk of response bias in that may have had a greater effect on participants in the intervention group whose frequent contact with the study may have made them more prone to wanting to be good experimental subjects. There are also challenges to identifying the aspects of the intervention that were responsible for the effectiveness. For instance, Intervention Group participants received more financial incentives than the Control Group which could have been responsible for differences rather than the engagement per se. There may also have been heterogeneity between aspects of the Intervention (as a hypothetical example, email messages may have improved effectiveness while mini-surveys may have reduced it). However, the qualitative component of the study (not reported here) suggested that participants were responding to the engagement aspects of the intervention.

## Supporting information

S1 Checklist CONSORT 2010 checklist of information to include when reporting a randomised trial*. (DOC)

S1 File Protocol. (DOCX)
