# Supplementary material for: Long-term engagement in smoking cessation campaign: A mixed methods randomized trial
Source: PLoS One. 2025 Jan 30;20(1):e0318160. doi: 10.1371/journal.pone.0318160 (PMC11781614; doi:10.1371/journal.pone.0318160)
Supplement: S1 File — (DOCX) [file pone.0318160.s002.docx]

**Study Protocol Information File**

The Canadian Cancer Society (CCS) and leaders in tobacco control across, including the Ontario Tobacco Research Unit (CCS) are working together to create, implement and evaluate a national tobacco cessation social marketing campaign. Evidence has shown that social marketing initiatives have positive effects on reducing the prevalence of smoking rates and consumption of tobacco [1,2]. The campaign will address a long-standing gap in the tobacco control ecosystem, using a combination of proven marketing techniques, including research, brand creation and awareness, advertising and incentives along with essential grassroots engagement and promotion. In this way the project seeks to reach and encourage quit attempts among the 2.6 million Canadians aged 35-64 who continue to smoke regardless of what stage of their cessation journey they are in. The campaign, innovatively executed in both English and French, and representative of the population of tobacco users, is designed to enhance awareness, knowledge, education and use of proven tobacco cessation mechanisms with the long-term goal of driving down tobacco use in pursuit of Canada’s goal of less than 5% tobacco prevalence rate by 2035. The overall goal of the proposed project is to develop and implement a comprehensive and synergistic tobacco cessation campaign to motivate quit attempts among Canadian adult tobacco users aged 35-64. Comprehensive in that it combines evidence-based strategies, messages and resources with the promotion of cessation supports; and, synergistic in that it works in coordination with other parts of Canada’s overall tobacco control strategy. The campaign will reach the tobacco user through four pillars of: awareness, knowledge/education, support and incentivization. The objectives of the project are: Objective 1: Increase adult tobacco users’ awareness of a) the consequences/harms of smoking; and b) the benefits of quitting (or reducing) tobacco use (I.e., health, social, financial) using evidence-based stigma reduction frameworks that are sensitive to culture, sex and gender. Objective 2: Increase adult tobacco users’ intentions to reduce smoking or to quit for good using inclusive education, compelling messaging, support resources and content that reaches tobacco users throughout the cessation process (Stages of Change) from precontemplation (not thinking of quitting) to maintenance (remaining a non-user). Objective 3: Increase quit attempts through the promotion of evidence-based cessation supports (E.g., Quitlines) and offering of incentives to encourage behaviour change (E.g., Quit and win contest). Smoking remains the number one cause of preventable disease and death in Canada [3] yet 2.6 million (35.3%) people aged 35-64 still smoke.[4] The current burden results in 45,000 deaths per year (18.4% of all deaths in Canada) and countless more experiencing tobacco-related illness and disease. To meet Canada’s Tobacco Strategy goal of a less than 5% tobacco prevalence rate by 2035, we need innovative, collaborative and easy-to access approaches that have mass reach. Robust efforts and collaboration are critical to make quitting the easy and obvious choice and this project is a huge step in the right direction. This is a large project with multiple components. OTRU will be involved in market research and evaluation activities. The market research phase will consist of a series of 3 focus groups and an online survey, which will also serve as a baseline measurement tool for the evaluation. Upon completion of market research, OTRU will lead a multi-pronged developmental and formative evaluation to capture the outcomes of the project and enable real-time learning during implementation. Results from both quantitative and qualitative data collection will inform opportunities for improvement and decisions about campaign refinement. Three overarching evaluation questions will be addressed: 1) what is the reach of the social marketing campaign? 2) how well does the campaign and each of its components resonate with the population and sub-groups (creative, website, cessation supports, incentives) 3) What are the outcomes of the campaign including awareness of the harms of tobacco use, knowledge of tobacco reduction, cessation benefits or supports, intentions to quit, quit attempts, successful quits?

REFERENCES 1. Durkin S, Brennan E, Wakefield M. Mass media campaigns to promote smoking cessation among adults: an integrative review. Tob Control. 2012 Mar 1;21(2):127–38. 2. Bala MM, Strzeszynski L, Topor-Madry R, Cahill K. Mass media interventions for smoking cessation in adults. In: Cochrane Database of Systematic Reviews [Internet]. John Wiley & Sons, Ltd; 2013 [cited 2016 Apr 15]. Available from: http://onlinelibrary.wiley.com/doi/10.1002/14651858.CD004704.pub3/abstract 3. Baliunas D, Patra J, Rehm J, Popova S, Kaiserman M, Taylor B. Smoking-attributable mortality and expected years of life lost in Canada 2002: conclusions for prevention and policy. (2007). Chronic Disease Canada, 27(4):154-62. PMID: 17623561. 4. Statistics Canada. Table 13-10-0096-10 Smokers, by age group DOI: <https://doi.org/10.25318/1310009601-eng>

BASLINE FOLLOW-UP SURVEYS A total of 3000 adult smokers in Canada (aged 35 to 65) will be recruited through paid, targeted ads on social media (i.e., Facebook, Instagram) to participate in online surveys The first online survey (baseline), implemented prior to social marketing roll-out, will serve the dual purpose of market analysis and creating baseline measures for key outcomes. Seven and 14-month follow-up surveys will assess exposure to elements of the social marketing campaign, resonance, and changes in knowledge, attitudes and behaviours in light of program components. Specifically, surveys will include questions related to knowledge (I.e., harms, benefits of quitting and cessation supports available), campaign awareness (aided and unaided), commercial tobacco use behaviour (e.g., smoking status, heaviness of smoking, quit attempts, intentions to quit, barriers to quitting etc..), campaign satisfaction/resonance and perceived campaign impacts. Socio-demographic questions will also allow for examination of differential impacts of the campaign by factors such as age, gender, race, SES, geography and official language. OTRU will randomly allocate survey participants into two groups of 1,500. The first group (intervention Group) will receive $200 and commit to review and respond to all program elements, in addition to completing the baseline, and two follow-up surveys. The second group (comparison group) will only complete the baseline and two follow-up surveys, and will not be asked to engage directly with the campaign materials. This group will receive $10 for each survey completed (total $30). Surveys will be administered online via RedCap, a secure, password protected data capture platform hosted on servers at University of Toronto. **At the moment, we are submitting the Baseline survey for approval. We will submit the other surveys at a later date.** FOCUS GROUPS There will be three rounds of focus groups, conducted with Canadians who smoke (aged 35-65 years old) and key informants who are stakeholders involved in the delivery of commercial tobacco control programs and service. The purpose of Round 1 is to gather the knowledge needed to develop a campaign strategy by learning about thoughts, motivations and experiences related to tobacco use; exploring intentions to change behaviour and barriers to change; identifying the benefits of quitting that users find most compelling; capturing the language and tone the audience prefers when discussing quitting; and learning about the audiences use of social channels and related activities. In Round 2, participants will be involved in the review of the creative elements and a prototype of the website. The third round of focus groups will be conducted to inform campaign refinement- branding, messaging, website, incentive element and overall marketing strategy. We will recruit focus group participants in two ways. First, adult smokers will be recruited through our baseline and follow-up online surveys. Survey respondents who express interest in participating in a focus group and who provide contact details will be invited to participate. Second, Key informants will be recruited through the advisory committee. The advisory committee will ask key stakeholders for permission to forward their contact information to the OTRU researchers, who will then invite key stakeholders to the focus group via email. Two staff members from OTRU will co-facilitate the focus groups and a French facilitator will be hired to conduct the French focus groups. Focus groups will be conducted virtually via Zoom in both English and French. To increase security we will: a) only send the meeting link just prior to the scheduled focus group so it can’t be shared ahead of time; b) not post the meeting link publicly; c) use passwords to access the meeting platform; c) complete a roll call at the beginning of the focus groups; and d) encourage all focus group participants to try their best to participate from a safe, private place where possible. Each round will consist of 5-8 focus groups, with approximately 5-8 participants per focus group. Focus groups will last 90 minutes and participants will receive a $40 e-gift card to thank them for their time and thoughts (only adult smokers will receive the e-gift card, key informants will not). Sessions will be audio recorded and transcribed by a third-party transcriptionist, for which confidentiality agreement will be created. After the interviews have been transcribed, the audio files will be immediately destroyed. A thematic interpretive analysis will be used whereby transcripts will coded by the researcher in NVIVO 12 using a coding structure based on the interview guides. Codes will be subsequently organized by theme. Themes will be compared across participant groups.

INTERVIEWS We will conduct a total of 30 telephone or virtual interviews with survey respondents. Interviews will occur at 7 and 14 months post-campaign launch (15 English-speaking and 15 French-speaking) to enrich understanding of campaign reach, resonance and impact (including unintended consequences). Interviews will last approximately 30-40 minutes. Verbal consent to participate and be audio-recorded will be obtained at the start of the interview. All interviews will be audio-recorded and transcribed verbatim via a third-party provider, for which confidentiality agreement will be created. After the interviews have been transcribed, the audio files will be immediately destroyed. A thematic interpretive analysis will be used whereby transcripts will coded by the researcher in NVIVO 12 using a coding structure based on the interview guides. Codes will be subsequently organized by theme. Themes will be compared across participant groups.
